# Neutral plasma-activated solution reverses polymyxin to inhibit polymyxin-resistant *Acinetobacter baumannii* by promoting the release of ROS and destroying the outer membrane *in vitro*

**DOI:** 10.1128/msystems.00784-25

**Published:** 2025-10-09

**Authors:** Wenjie Yuan, Ting Yu, Xinxing Yang, Tao Lin, Tingting Guo, Xiaobin Wang, Guocai Li, Kaizheng Gong, Weili Liu

**Affiliations:** 1 Institute of Translational Medicine, School of Medicine, Yangzhou University 737447, Yangzhou, China; 2 Department of Critical Care Medicine, Affiliated Hospital of Yangzhou University, Yangzhou University 38043, Yangzhou, China; 3 Department of Laboratory Medicine, Affiliated Hospital, Yangzhou University 38043, Yangzhou, China; 4 School of Petroleum Engineering and Natural Gas Engineering, Changzhou University 12412, Changzhou, China; 5 Department of Cardiology, Affiliated Hospital of Yangzhou University, Yangzhou University 38043, Yangzhou, China; University of Technology Sydney, Sydney, New South Wales, Australia

**Keywords:** *Acinetobacter baumannii*, neutral plasma activated solution, polymyxin B, combination

## Abstract

*Acinetobacter baumannii* is a prominent pathogen linked to ventilator-associated pneumonia (VAP) and has demonstrated widespread multidrug resistance globally. While the exploration of synergistic antibiotic combinations is increasingly viewed as an innovative treatment strategy, the antibacterial potential of plasma activated water (PAW) in neutralizing formulations remains underdeveloped. This study investigates the synergistic interactions between neutral plasma activated water (NPAW) and polymyxin B against multidrug-resistant strains of *A. baumannii*. We evaluated the antibacterial activity of the combination of NPAW and polymyxin B against polymyxin-resistant *A. baumannii* strains both *in vitro* and *in vivo*, further exploring the underlying mechanisms of synergy. Through checkerboard assay and time-kill studies, we demonstrated that the combination of NPAW and polymyxin B exhibited a synergistic effect against polymyxin-resistant *A. baumannii*. In the mouse pneumonia model, we confirmed that the combined treatment significantly reduced bacterial colonization in the lungs. Mechanistic studies indicated that NPAW enhances the bactericidal activity of Polymyxin B by promoting the release of reactive oxygen species (ROS). When used together, NPAW and polymyxin B decreased the production of intracellular ATP and membrane potential and compromised outer membrane integrity. In conclusion, the synergistic interactions between these agents may enable the use of lower concentrations of polymyxin B in treating *A. baumannii* infections, thereby minimizing dose-dependent side effects and providing a novel therapeutic option for managing these infections.

**IMPORTANCE:**

Polymyxin-resistant *Acinetobacter baumannii* poses a global threat as last-line therapies fail. We demonstrate that neutral plasma activated water (NPAW), a reactive oxygen species-rich non-antibiotic agent, synergizes with polymyxin B to overcome resistance. Mechanistically, NPAW disrupts membrane integrity, depletes ATP, and amplifies oxidative stress, enhancing polymyxin B’s bactericidal activity and reducing lung bacterial burdens in mice. This synergy enables lower polymyxin B doses, a critical advance for treating ventilator-associated pneumonia.

## INTRODUCTION

The escalating global antimicrobial resistance (AMR) crisis poses a significant threat to healthcare systems, particularly due to multidrug-resistant Gram-negative pathogens (1, 2). As an opportunistic pathogen, *Acinetobacter baumannii* has been designated by the World Health Organization as a carbapenem-resistant critical priority pathogen, given its capacity to cause life-threatening ventilator-associated pneumonia, bloodstream infections, and urinary tract infections (3, 4). Epidemiological studies reveal that over 70% of clinical isolates exhibit multidrug resistance, with mortality rates exceeding the 50% threshold for intensive care unit (ICU)-acquired infections (1). The resistance mechanisms primarily stem from its intrinsic impermeable outer membrane, overexpression of multidrug efflux pump systems, and antibiotic-modifying enzymes (5). Notably, its ability to form biofilms and adapt metabolically to microenvironmental conditions exacerbates therapeutic challenges, driving recurrent infections and persistent resistance evolution (6).

Despite substantial global investment in research and development, few novel antibiotics targeting Gram-negative infections have been approved in the past three decades (7, 8). This critical gap underscores the urgent need for innovative antimicrobial strategies. Repurposing existing pharmaceuticals to enhance antibiotic efficacy, particularly through antibiotic-adjuvant combination therapies, has emerged as a cost-effective solution to mitigate the AMR crisis (9–11). Such synergistic approaches leverage multi-target mechanisms to not only potentiate antibiotic activity and prolong clinical utility but also reduce treatment-related toxicity through dosage optimization.

Current research demonstrates that plasma-activated water (PAW), synthesized through non-thermal plasma (NTP; ≤340 K) treatment of distilled water, exhibits broad-spectrum antimicrobial efficacy (12–14). Plasma systems are principally categorized into thermal plasma (≥15,000 K) and NTP (15), with NTP demonstrating superior biocompatibility through reactive oxygen/nitrogen species (RONS)-mediated pathogen inactivation (16). Notably, PAW, a novel aqueous disinfectant produced via NTP treatment, has attracted significant attention for its capacity to stabilize RONS components, including reactive nitrogen species (·NO, ONOO⁻, NO₂·) and reactive oxygen species (ROS) (·OH, H₂O₂, O₂⁻) (17). However, conventional PAW’s antimicrobial activity remains constrained to acidic conditions (pH < 5) (18). Our study shows that the development of neutral PAW (NPAW) and systematically elucidates its synergistic antibacterial mechanisms with conventional antibiotics for the first time.

We provide the first experimental evidence of a dual mechanism underlying NPAW-polymyxin B synergy: (i) outer membrane destabilization via transmembrane potential collapse and (ii) amplification of polymyxin-mediated oxidative stress. This combinatorial strategy not only overcomes the pH limitations of conventional PAW but also establishes a novel inhalation-based therapeutic paradigm for pulmonary infections.

## MATERIALS AND METHODS

### Preparation of plasma-activated water

**Fig 1:**
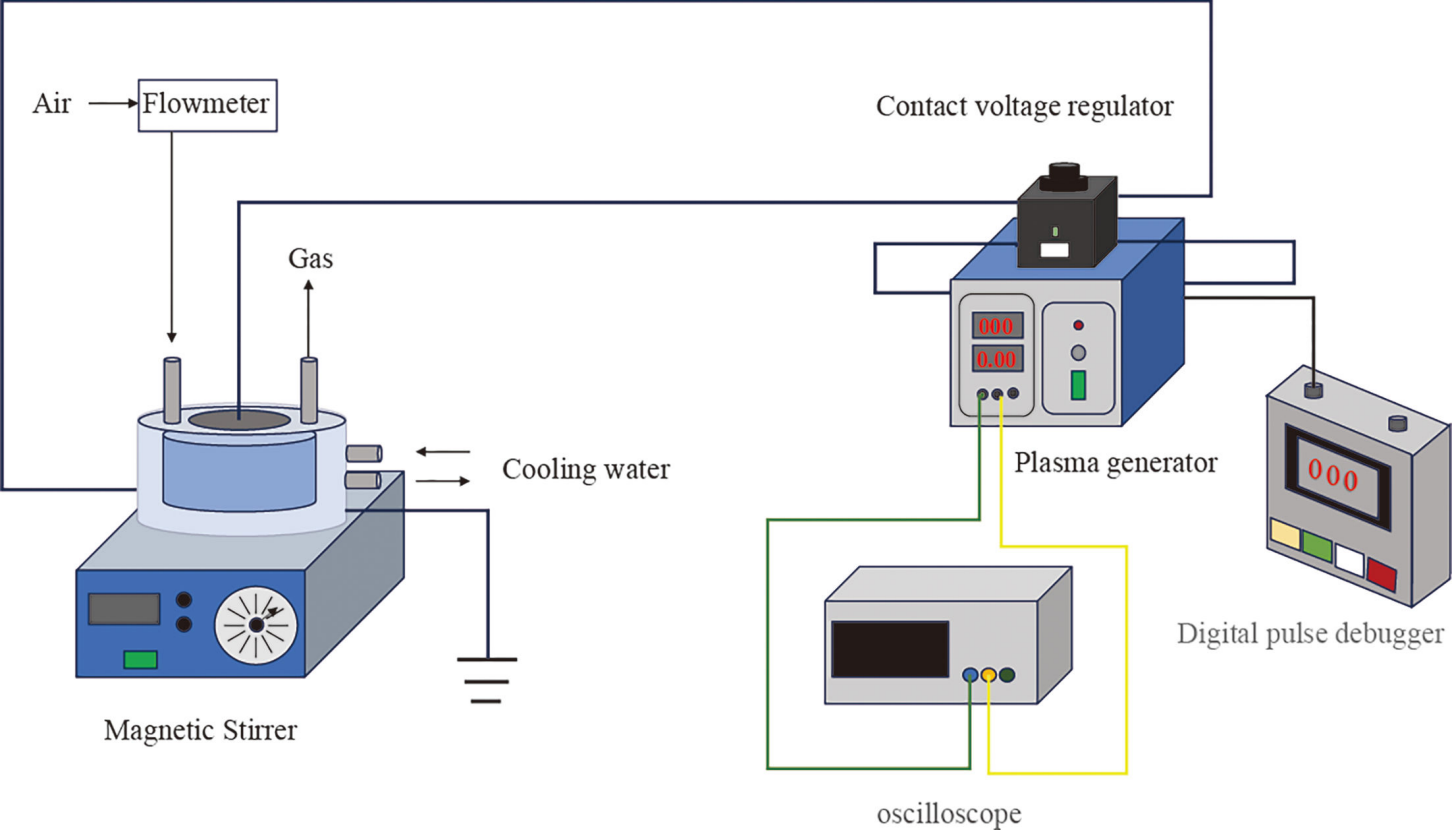
Preparation of PAW. The plasma device for preparing activated water consists of a high-voltage planar electrode, a grounded electrode, and a dielectric layer.

As shown in Fig. 1, the plasma device consisted of a high-voltage plane electrode, a grounded electrode, and a dielectric layer sandwiched between the two electrodes. Natural air was employed as the working gas, and the peak-to-peak voltage was 60 kV. Deionized water (30 mL) in a quartz dish was located beneath the plasma. The depth of the deionized water was approximately 2 mm, whereas the air gap between the plasma and the liquid surface was approximately 8 mm. The duration time for plasma irradiation was 25 min. After exposure to the plasma, the pH of PAW was measured using a pH meter, which was approximately 1.0–1.5. The pH of PAW was adjusted by a 5% NaHCO_3_ solution, and the pH of NPAW was adjusted to 7.4 ± 0.1.

### Bacterial strains

In this study, three non-clonal clinical *A. baumannii* strains AB13, AB301, and AB304 obtained from the Affiliated Hospital of Yangzhou University, along with the standard strain ATCC 19606, were utilized. AB13 is a polymyxin-sensitive strain, and AB301 and AB304 are polymyxin-resistant strains. The strains AB13R and ATCC 19,606R (polymyxin B resistance) were derived from AB13 and ATCC 19606 through continuous subculture under a polymyxin B concentration gradient.

### MIC determination and synergy test

The antimicrobial susceptibility profiles of *A. baumannii* to polymyxin B and NPAW were quantitatively assessed through triplicate minimum inhibitory concentration (MIC) determinations, performed in strict accordance with Clinical and Laboratory Standards Institute (CLSI) broth microdilution methodology. Briefly, serial twofold dilutions of antimicrobial agents in Mueller-Hinton broth (MHB) (Hopebio, Qindao, China, HB6231) were combined with standardized bacterial suspensions in 96-well microplates, achieving final inoculum densities of 5 × 10⁵ CFU/mL. Following 18–24 h incubation at 37°C, MIC endpoints were defined as the lowest drug concentration demonstrating complete growth inhibition.

To assess the synergistic effects between NPAW and antibiotics, a standard checkerboard broth microdilution test was carried out. All the tests were performed in triplicate. For the checkerboard assay, the experiments were performed three times.

### Time-dependent killing curve

*A. baumannii* cultures were propagated in MHB through sequential dilution (1:100) of overnight suspensions, followed by incubation at 37°C with 200 rpm until mid-log phase (optical density at 600 nm [OD600] = 0.5). The cultures were subsequently exposed to either polymyxin B or NPAW alone or their combination. At predetermined time intervals, the samples underwent 10-fold serial dilution in phosphate-buffered saline (PBS, pH 7.4). Viable bacterial counts were quantified via spot-plating methodology, wherein 10 µL aliquots of appropriate dilutions were inoculated onto LB agar plates and incubated aerobically (37°C, 18–24 h). Experimental procedures were executed with three independent biological replicates.

### LIVE/DEAD staining assay

Bacteria cultured in the logarithmic growth phase were diluted to 5 × 10^6^ CFU/mL in MHB, and the diluted inoculum was added to a 24-well plate with 14-mm glass climbing tablets (Nest, Wuxi, China). After incubation for 24 h at 37°C, the tablets were washed three times with 0.9% NaCl and stained with SYTO9 and PI (Fushenbio, Shanghai, China) for 15 min in the dark. The tables were observed under a laser confocal scanning microscope (Nikon, Japan), and the images were analyzed using Imaris software.

### Safety assessment

Hemolysis assay of polymyxin B in the presence of NPAW was evaluated as follows. Then, 8% sheep blood cells (Solarbio, Beijing, China, S9450) that were prepared from fresh sterile defibrinated sheep blood were treated with various concentrations of PAW alone or in combination with polymyxin B (2 µg/mL). Triton X-100 at 1% was used as a positive lysis control. The plate was incubated at 37°C for 1 h. After that, samples were centrifuged (1,000×*g* for 10 min at 4°C) to remove the intact erythrocytes, and the supernatant was transferred to a new 96-well plate. The absorbance of released hemoglobin was measured at OD570, and the percentage of hemolysis was calculated.

The cytotoxic effects on MLE-12 cells were performed by Cell Counting Kit-8 (CCK-8) assay, following the manufacturer’s instructions (Beyotime, Shanghai, China, C0038). The MLE-12 cells were seeded into a 96-well plate at a density of 2 × 10^3^ cells per well and cultured to 90% confluency. Subsequently, the cells were treated with PAW alone or in combination with polymyxin B (2 µg/mL) (Solarbio, Beijing, China, P8350) for 12 h, with three replicates for each concentration. Following the 12-h incubation period, 10 µL of CCK-8 solution was added to per well, and the absorbance was measured at 450 nm to assess the cytotoxic effect of NPAW.

### Mouse pneumonia model

Female BALB/c mice (8–12 weeks old; 20–24 g) were obtained from the Comparative Medicine Center of Yangzhou University and acclimatized under controlled environmental conditions for 7 days before experimentation. Neutropenia was induced through intraperitoneal administration of cyclophosphamide (150 mg/kg on day −4 and 100 mg/kg on day −1 relative to infection) in 36 mice. Pulmonary infection was established via intratracheal instillation of 100 µL bacterial suspension (2.0 × 10⁷ CFU/mouse). Following a 2-h post-infection period, animals were randomly assigned to six groups (*n* = 6 per group) implementing distinct intervention protocols. The therapeutic regimens consisted of intraperitoneal (i.p.) drug administration and 20-min inhalation (Inh) therapy, delivered at 12 h intervals across four consecutive treatment cycles. The groups included (i) Control: saline i.p. and saline Inh, (ii) NPAW Inh: saline i.p. +NPAW Inh, (iii) PMX-B i.p.: polymyxin B i.p. (5 mg/kg) + saline Inh, (iv) PMX-B Inh: saline i.p. + polymyxin B Inh (5 mg/mL in saline), (v) NPAW Inh + PMX B i.p.: polymyxin B i.p. + NPAW Inh, (vi) NPAW Inh + PMX B Inh: saline i.p. + polymyxin B (5 mg/mL in NPAW) and NPAW Inh. Lungs were aseptically excised, weighed, and homogenized in PBS for serial dilution plating. Bacterial burden was quantified as colony-forming units (CFU) per gram of lung tissue.

### Membrane potential assay

To measure the changes in membrane potential with DiSC3(5) (Beyotime, Shanghai, China, ANS-AS-84923). Bacterial cultures were harvested by centrifugation, washed twice in sterile PBS, and resuspended in 5 mM HEPES buffer (pH 7.0, supplemented with 5 mM glucose) to an OD600 = 0.5. Then, the samples were pretreated for 30 min at 37°C with the following: (i) NPAW (5%–40%), (ii) polymyxin B (2 µg/mL), (iii) polymyxin B (2 µg/mL) + 20% NPAW, or (4) no treatment (negative control). Following pretreatment, DiSC3(5) (Yuanye Bio-Technology, Shanghai, China) was added to a final concentration of 0.5 µM and incubated for 15 min in the dark. Fluorescence intensity was quantified using a fluorescence spectrophotometer (excitation: 622 nm, emission: 670 nm), with signal attenuation proportional to membrane depolarization. All experiments included triplicate biological replicates.

### Outer membrane permeability assessment

To evaluate outer membrane integrity using the hydrophobic fluorescent probe 1-N-phenylnaphthylamine (NPN). Bacterial suspensions were treated for 1 h at 37°C with different concentrations of NPAW (0%–40%) or polymyxin B (0–1 μg/mL) or its combination with 20% NPAW. Following treatment, NPN (Sigma-Aldrich) was added to a final concentration of 10 µM and incubated for 5 min in the dark. Fluorescence intensity was immediately quantified using a microplate reader with 350-nm excitation and 420nm emission wavelengths.

### ROS measurement

To measure the generation of ROS (Beyotime, Shanghai, China, S0033S) using a fluorescent probe, DCFH-DA (2′,7′-dichlorodihydrofluorescein diacetate; Sigma-Aldrich, USA). Bacterial cultures were harvested by centrifugation, washed twice in sterile PBS, and standardized to 1 × 10^7^ CFU/mL in PBS. Aliquots were treated with different concentrations of NPAW or polymyxin B, or their combination. After centrifugation, the bacteria were resuspended in 0.9% NaCl, and DCFH-DA was added to a final concentration of 2 µM and incubated for 30 min at 37°C in the dark. Fluorescence intensity was quantified immediately using a fluorescence microplate reader with 488-nm excitation and 530nm emission wavelengths.

### Intracellular ATP level determination

Intracellular ATP levels of bacteria were determined using an Enhanced ATP Assay Kit (Beyotime, Shanghai, China, S0027). The cells were cultured overnight and resuspended in PBS, then treated with NPAW (20%) or polymyxin B (2 µg/mL) or their combination with 20% NPAW for 0–2 h. After centrifugation, the bacterial precipitates were treated with lysozyme, and the supernatants were collected for subsequent determination. The test solutions were added to the 96-well plates and incubated at room temperature for 5 min. The luminescence was detected by a multi-function microplate reader.

### Resistance development analysis

Due to the sensitivity of the *A. baumannii* isolate, ATCC19606, to polymyxin B, it was employed further in the resistance development studies. ATCC 19606, after culture at 37°C for 24 h, was diluted in 1:100 MHB media supplement with 0.25× MIC of polymyxin B or polymyxin B plus 20% NPAW, which was then cultured again at 37°C for 24 h while determining the MIC by twofold serial dilutions in 96-well microtiter plates. Furthermore, the cultures were again diluted in 1:100 MHB media containing 0.25× MIC of drugs for a total of 28 passages containing ATCC19606, inducing polymyxin-resistant strains along with the calculation of the fold increase in subsequent polymyxin B MIC relative to the initial MIC.

### Statistical analysis

Data were presented as mean ± standard deviation (SD) of three replications. Mean values between groups were compared using the two-tailed Student’s *t*-test. All statistical analyses were performed using GraphPad Prism version 8.4.2, with a *P* value < 0.05 considered significant.

## RESULTS

### Synergistic activity of NPAW in combination with polymyxin B

**Fig 2:**
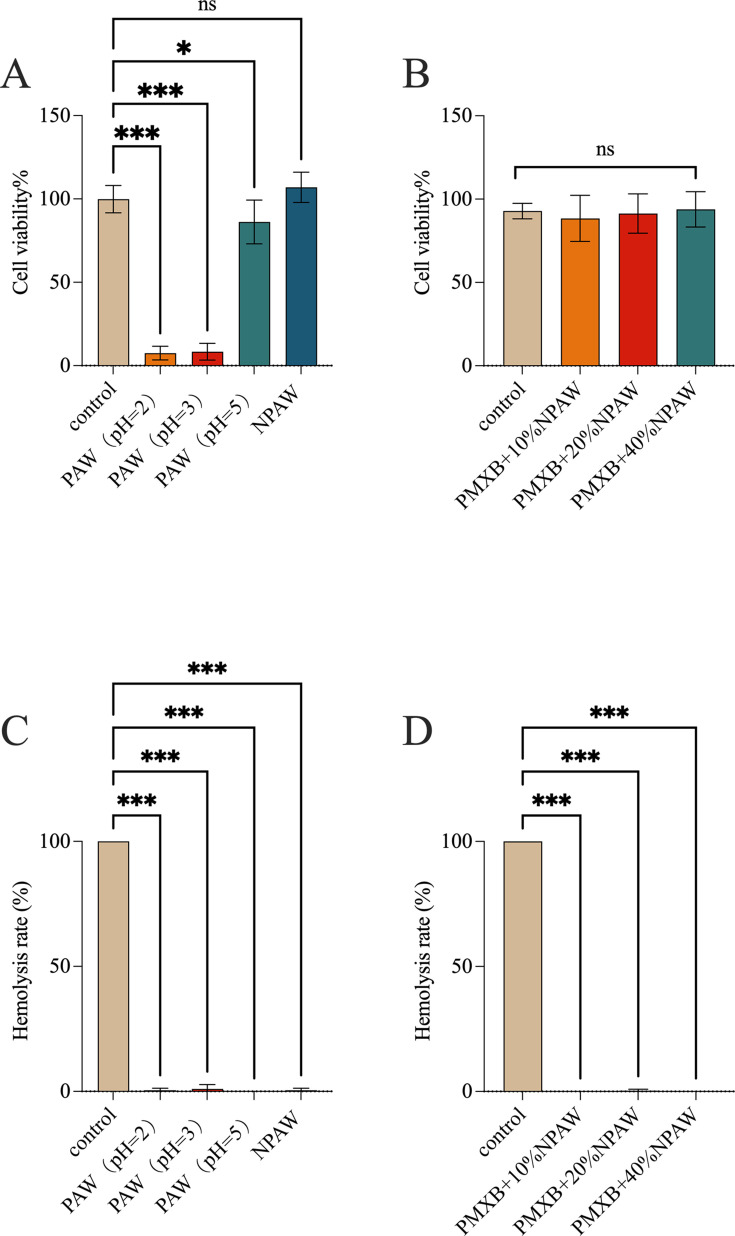
Safety assessment of PAW alone or in combination with polymyxin B (2 µg/mL). Cell viability of MLE-12 cells was analyzed using the CCK-8 assay in different PH PAW alone (**A**) and NPAW in combination with polymyxin B (**B**) (*n* = 8). Hemolytic activity in different pH PAW alone (**C**) and NPAW in combination with polymyxin B (**D**) (*n* = 4). Error bars denote the mean ± SD of three replicates. ns, *P* > 0.05, *P < 0.05, ***P < 0.001—one-way ANOVA with Dunnett *post hoc* test.

To evaluate the potency of NPAW as an antibacterial, we first measured the content of PAW at different pH levels on cell viability. The results showed that PAW has good antimicrobial activities at a pH of 2 and 3 (Table 1). However, PAW at a pH of 2 and 3 has high cytotoxicity (Fig. 2A), and the effect of PAW on red blood cells was negligible (<5%). When PAW was at a pH of 7.4 (NPAW), it did not affect the growth of bacteria (Table 1) and had no cytotoxicity to MLE cells (Fig. 2A) and had negligible effects on the hemolysis of red blood cells. The results revealed the lack of direct antimicrobial activity of NPAW alone against the AB301, AB304, AB13, AB13R, ATCC 19606, and ATCC 19606R strains.

**TABLE 1:** The percentage of PAW with different pH to achieve antimicrobial activities^*a*^

Strains	PAW pH = 2.0	PAW pH = 3.0	PAW pH = 5.0	NPAW pH = 7.4
AB301	5%	10%	–	–
AB304	10%	20%	–	–
ATCC19606	2.5%	10%	–	–
ATCC19606R	2.5%	10%	–	–
AB13	5%	20%	–	–
AB13R	5%	20%	–	–

^*a*^ PAW, plasma-activated water; “–,” bacterial growth reaches 100%.

To determine the efficacy of NPAW in combination with antibiotics in inhibiting pandrug-resistant *A. baumannii*, the synergistic antimicrobial activity of NPAW combined with several antibiotics was investigated by checkerboard broth microdilution assays. The activities of 11 antibiotics (ticarcillin, ampicillin, cefepime, ceftazidime, meropenem, levofloxacin, amikacin, gentamicin, minocycline, tigecycline, and polymyxin B) with NPAW against the pandrug-resistant *A. baumannii* isolate AB304 are shown in Table 2. Through primary screening, NPAW showed a synergistic effect with polymyxin B against AB304.

**TABLE 2:** Synergistic activities of NPAW in combination with different antibiotics against pandrug-resistant AB304

Antibiotic	MIC (μg/mL)	MIC with 20% NPAW (μg/mL)	Fold change
Ticarcillin	≥1,024 (R)	≥1,024 (R)	1
Ampicillin	≥1,024 (R)	≥1,024 (R)	1
Cefepime	≥1,024 (R)	≥1,024 (R)	1
Ceftazidime	≥1,024 (R)	≥1,024 (R)	1
Meropenem	256 (R)	256 (R)	1
Levofloxacin	128 (R)	16 (R)	8
Amikacin	≥1,024 (R)	≥1,024 (R)	1
Gentamicin	≥1,024 (R)	512 (R)	2
Minocycline	32 (R)	16 (R)	2
Tigecycline	16 (R)	8 (I)	2
Polymyxin B	32 (R)	0.5 (S)	64

### Synergistic activity of NPAW and polymyxin B

To explore the interaction of NPAW and polymyxin B, a checkerboard assay was performed for the wild-type and laboratory-generated polymyxin-resistant *A. baumannii*. Checkerboard assays revealed the obvious dose-dependent synergistic activity of NPAW with polymyxin B on the AB301, AB304 wild-type polymyxin-resistant strains, and ATCC19606R, AB13R laboratory-generated polymyxin-resistant strains (Table 3). These results suggested that polymyxin-resistant *A. baumannii* strains have recovered or enhanced sensitivity to polymyxin B when combined with NPAW. Among them, 20% and 40% NPAW had the most significant effect when combined with polymyxin. To evaluate the safety of NPAW as an atomization agent, we performed toxicity tests in MLE-12 cells. The results showed the NPAW combination with polymyxin B had no cytotoxicity to MLE-12 cells and had negligible effects on the hemolysis of red blood cells.

**TABLE 3:** Polymyxin B MIC of wild-type and laboratory-generated polymyxin-resistant strains when treated with different concentrations of NPAW

Strain	Polymyxin B MIC (μg/mL) at different NPAW concentrations						
	Only polymyxin B	Npaw (10%)		Npaw (20%)		Npaw (40%)	
		MIC	Fold change	MIC	Fold change	MIC	Fold change
AB 301	16	0.5	32	0.25	64	0.25	64
AB 304	32	1	32	0.5	64	0.5	64
ATCC19606	0.5	0.5	1	0.5	1	0.5	1
ATCC19606R	1,024	0.5	2,048	0.5	2,048	0.5	2,048
AB 13	1	1	1	0.5	2	0.5	2
AB 13R	128	1	128	0.5	256	0.5	256

**Fig 3:**
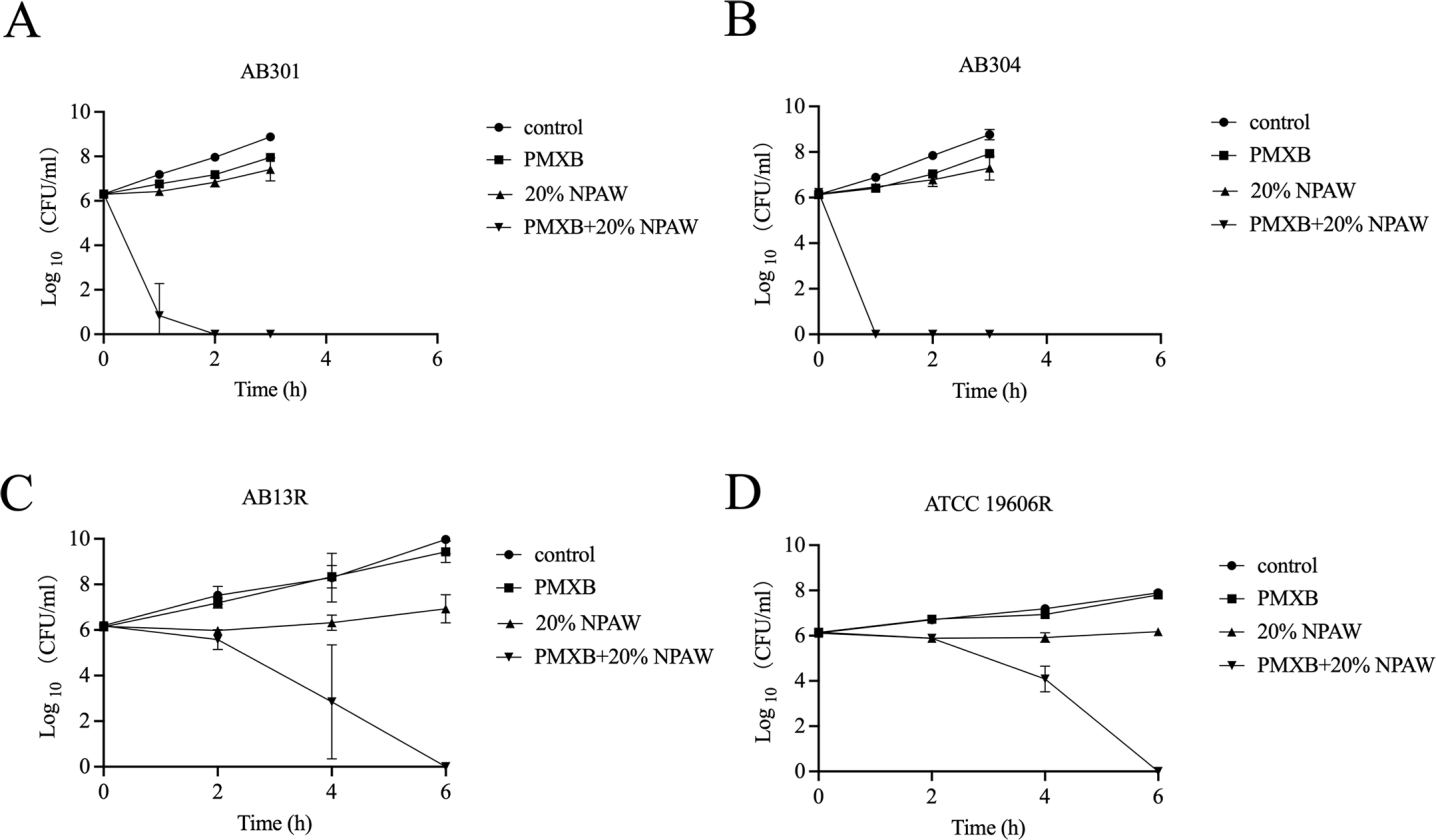
Time-dependent killing of *A. baumannii* using polymyxin B alone or in combination with 20% NPAW. Time-dependent killing curve of *A. baumannii* AB301 (**A**), AB304 (**B**), AB13R (**C**), and ATCC 19606R (**D**) using polymyxin B (2 µg/mL) alone or with 20% NPAW. Data are presented as mean ± SD.

**Fig 4:**
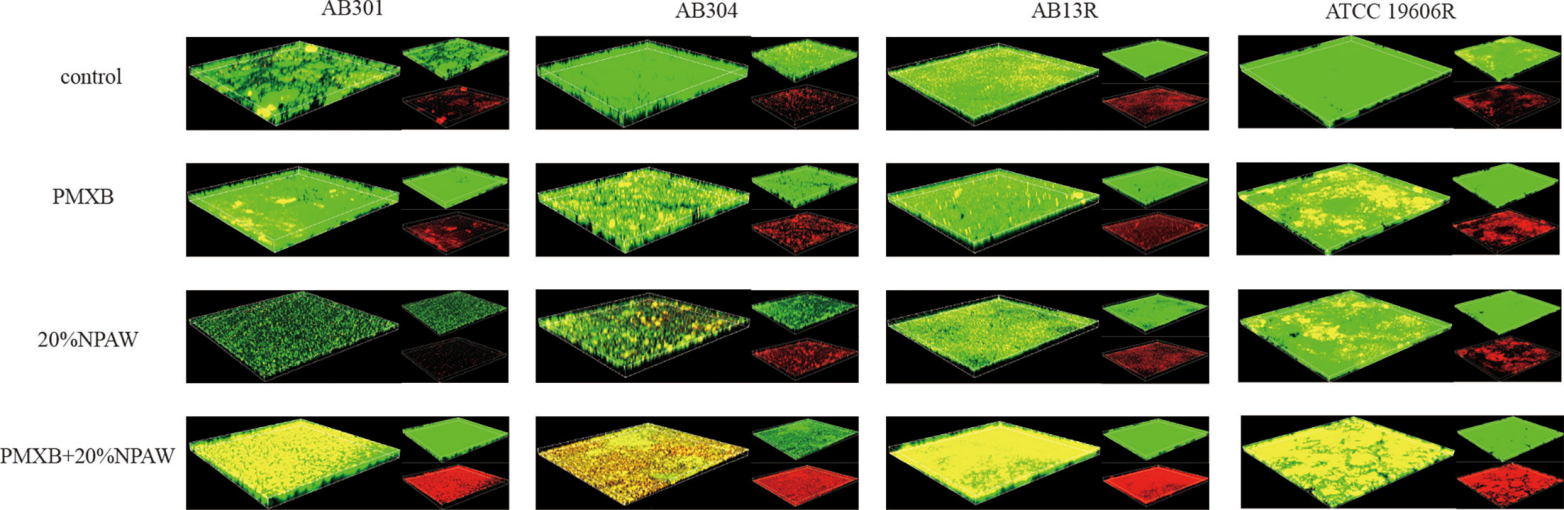
LIVE/DEAD staining of *A. baumannii* using polymyxin B alone or in combination with 20% NPAW (green and red fluorescence represent live bacteria and dead bacteria, respectively).

To further confirm the synergism of NPAW with polymyxin B, time-kill assays were carried out with the four polymyxin-resistant strains. The polymyxin B concentration used corresponded to the clinical breakpoint for susceptibility (2 µg/mL), while the NPAW concentration was 20%, which could recover or enhance sensitivity to polymyxin B (Table 3). As shown in Fig. 3 and 4, neither NPAW nor polymyxin B monotreatment killed polymyxin-resistant *A. baumannii*. However, the combination can be reduced to undetectable levels. The above results showed that NPAW drastically enhanced the effect of polymyxin B against polymyxin-resistant *A. baumannii*.

### Therapeutic efficacy of polymyxin B in combination with NPAW in the mouse pneumonia model

**Fig 5:**
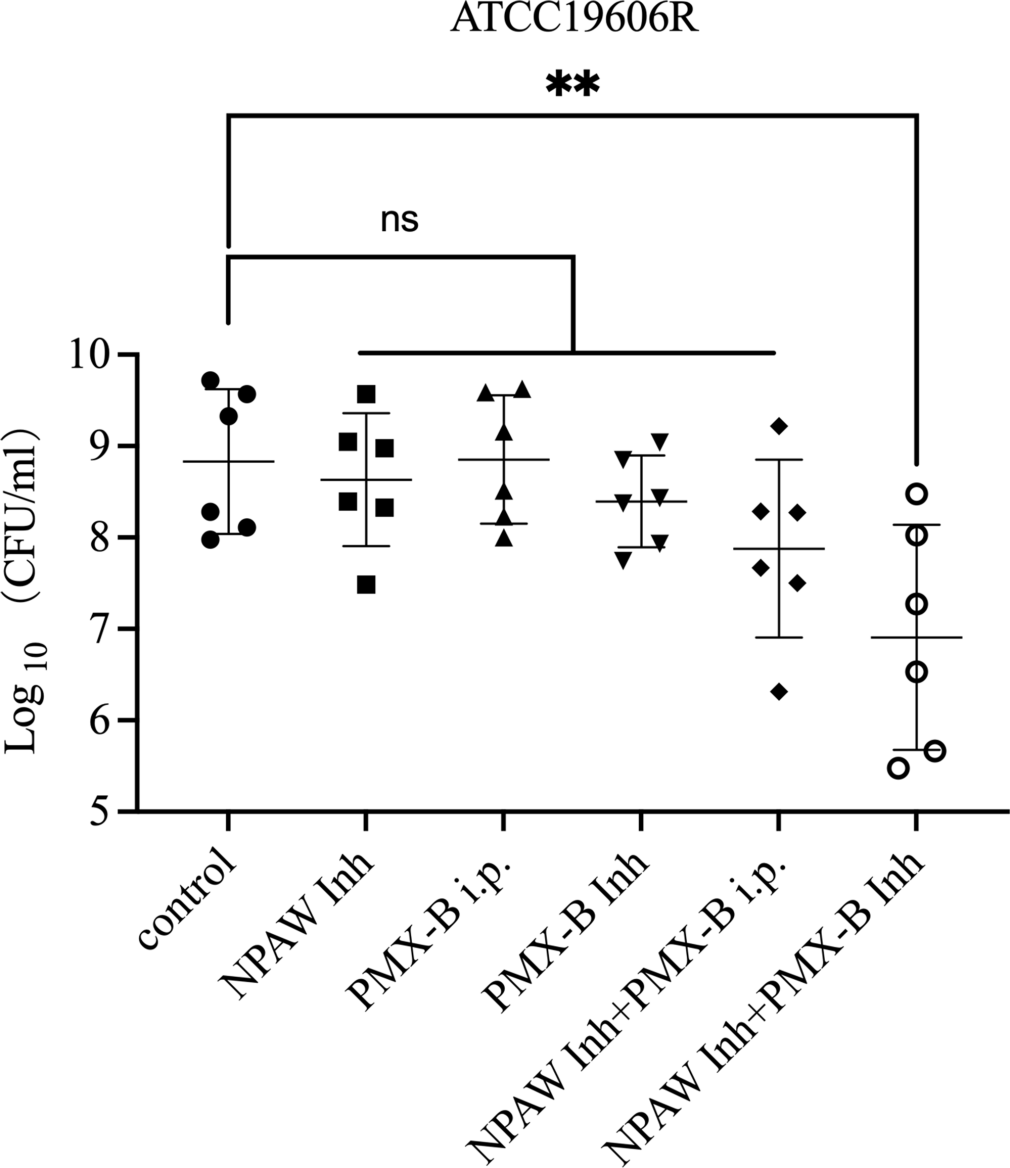
*A. baumannii* burden in the lungs. The control group received NS i.p. and NS Inh, the NPAW Inh group received NS i.p. and NPAW Inh, the PMX-B i.p. group received 5 mg/kg PMX-B i.p. and NS Inh, the PMX-B Inh group received NS i.p. and 5 mg/mL PMX-B Inh, the NPAW Inh + PMX B i.p. group received 5 mg/kg PMX-B i.p. and NPAW Inh, and the NPAW Inh + PMX B Inh group received NS i.p. and PMX-B 5 mg/mL dissolved in 100% NPAW Inh. ***P < 0.01 was determined using the Student’s *t*-test (*n* = 6).

*A. baumannii* is a common pathogen of hospital-acquired pneumonia, so to determine the potential use of NPAW atomization therapy in clinical situations, we investigated the *in vivo* efficacy of NPAW inh combined with polymyxin B in a pneumonia model infected with polymyxin-resistant *A. baumannii*. In preliminary experiments, we found that the NPAW inh + PMX B Inh treatment group showed reduced *A. baumannii* densities in the lungs compared with the untreated or NPAW inh or PMX-B i.p. or PMX-B inh alone groups (Fig. 5).

### NPAW reduces membrane potential

**Fig 6:**
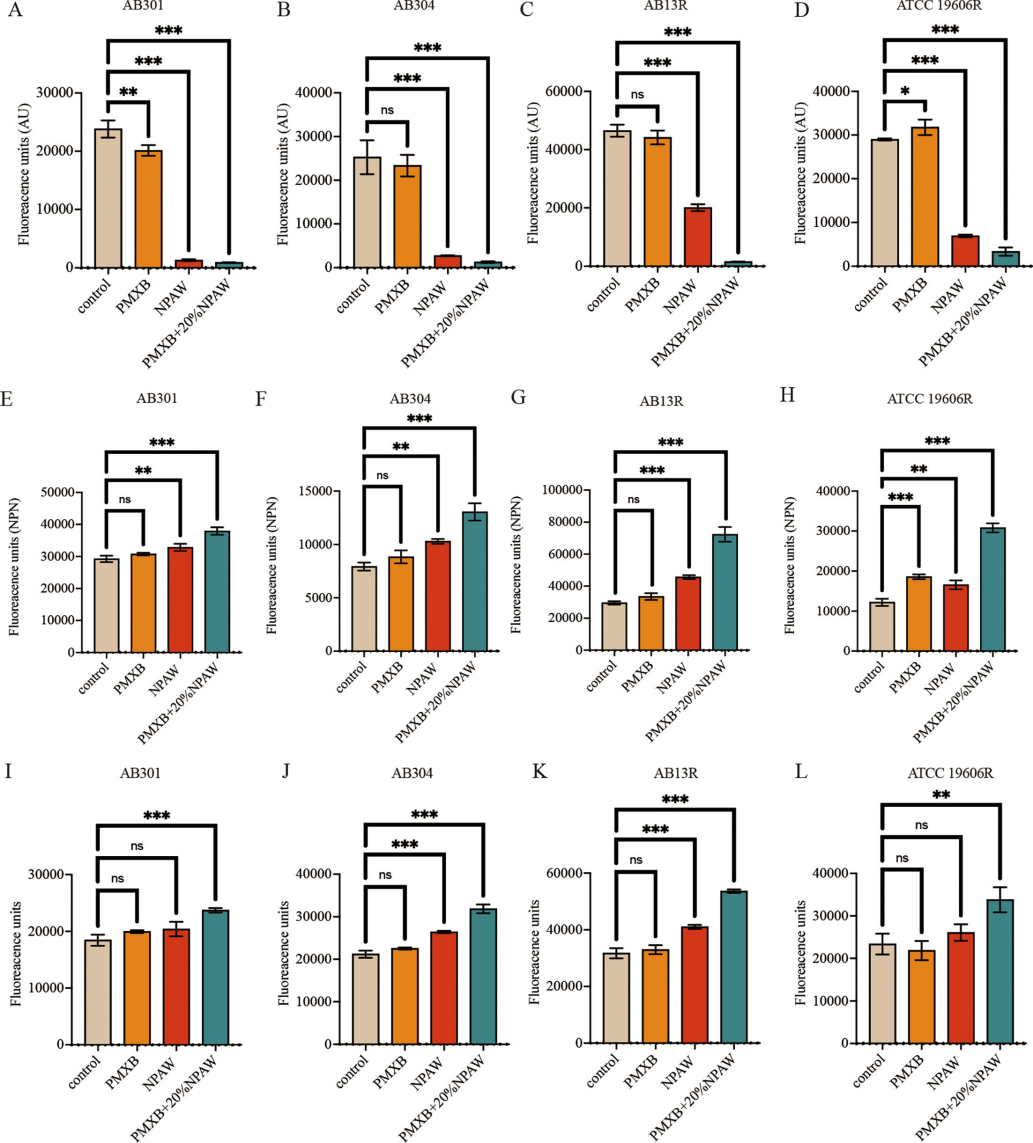
The effect of NPAW on the membrane potential, permeability of the outer bacterial membrane, and ROS production of *A. baumannii*. The membrane potential (**A–D**), permeability of the outer bacterial membrane (**E–H**), and ROS production (**I–L**) were treated with polymyxin B (2 µg/mL) and NPAW in AB301, AB304, AB13R, and ATCC 19606R. Data are presented as mean ± SD of three replicates. ns, *P* > 0.05, *P < 0.05, ***P < 0.001—one-way ANOVA with Dunnet *post hoc* test.

We measured the changes in membrane potential with DiSC_3_(5). Figure 6A through D shows that the addition of NPAW significantly reduced the membrane potential. A membrane potential assay was performed to determine the efficacy of the NPAW combination with polymyxin B in reducing membrane potential, confirming that NPAW can indeed reduce membrane potential. However, polymyxin B itself does not affect membrane potential (Fig. 6A through D).

### NPAW destroys the outer membrane

We further measured the changes in cell membrane permeability after NPAW treatment. 1-N-phenylnaphthylamine (NPN) was used to study the outer membrane permeability. Figure 6E through H shows that the addition of NPAW significantly increased outer membrane permeability of laboratory-generated polymyxin-resistant strains in a concentration-dependent manner.

### Effect of NPAW on the production of ROS and the content of intracellular ATP

**Fig 7:**
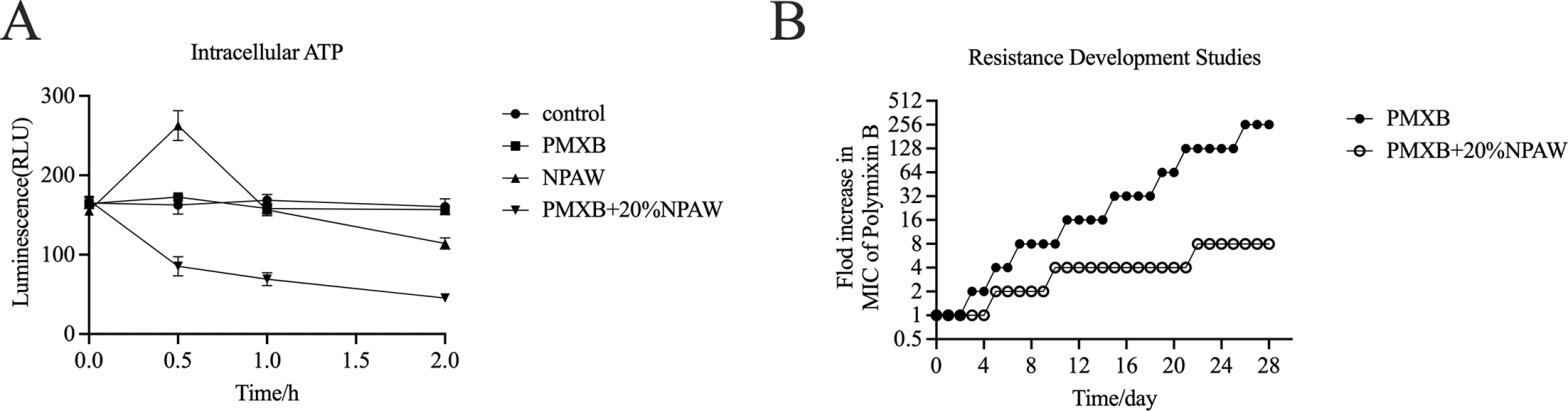
The intracellular ATP levels and NPAW prevent the evolution of antibiotics-elicited resistance of *A. baumannii*. (**A**) Intracellular ATP in ATCC 19606R treated with polymyxin B (2 µg/mL) and NPAW. (**B**) Resistance acquisition curves during serial passage with polymyxin B (2 µg/mL) and 20% NPAW or their combination against ATCC19606. The data shown here are the mean ± SD of three replicates.

The membrane damage is related to the production of ROS and affects the content of ATP, and the bacteria will produce oxidative damage under antibiotic stress. Therefore, we speculate that NPAW may increase oxidative damage. As shown in Fig. 6I through L, the ROS levels of *A. baumannii* were detected, and it was observed that the polymyxin B monotherapy group did not display any change of fluorescent intensity when compared with the control and NPAW monotherapy groups that showed an increased ROS production with higher concentrations, respectively. The effect of NPAW on intracellular ATP levels was determined by the luciferase bioluminescence method. As shown in Fig. 7A, the NPAW combination with polymyxin B significantly decreased the intracellular ATP level.

The above results showed that the synergistic effect of NPAW and polymyxin B is associated with ROS production and intracellular ATP levels.

### Resistance development studies

To further understand the ability of NPAW to inhibit the development of antibiotic resistance, resistance development studies were conducted. A total of 28 passages of ATCC 19606 with polymyxin B sub-MIC (0.25× MIC) in 20% NPAW’s presence and absence were performed. As it was observed that the speed of polymyxin B resistance development of the polymyxin B alone group was faster than the combination group (Fig. 7), which indicated that the combination of NPAW and polymyxin B could delay the emergence of polymyxin B resistance.

## DISCUSSION

The ongoing emergence of multidrug-resistant (MDR) *A. baumannii*, particularly strains resistant to carbapenems and polymyxins, is a significant concern (2). Recent surveillance data indicate that in the ICU, 45%–68% of carbapenem-resistant *A. baumannii* (CRAB) strains exhibit resistance to polymyxin B (19). These alarming statistics highlight the urgent need for strategies that leverage adjunctive therapies to restore the efficacy of existing antibiotics.

Historically, traditional antibiotics have been the cornerstone of bacterial eradication. However, rising failure rates are now attributed to complex resistance mechanisms, including impermeable outer membranes, overexpression of efflux pumps, and enzymatic drug inactivation (4, 20, 21). This crisis necessitates innovative strategies that bypass existing resistance pathways while minimizing collateral toxicity. Our study addresses this dual challenge by pioneering the use of NPAW as an adjunct to polymyxin B, demonstrating synergistic effects against polymyxin-resistant *A. baumannii* through distinct mechanisms.

Current adjunctive therapies for *A. baumannii* resistance include immunotherapeutic approaches aimed at enhancing host immune function to support antibiotic efficacy (22, 23), synergistic antibiotic combination strategies (24, 25), and novel biomaterials combined with physical therapies (9), such as nanoparticle drug delivery systems and phage-antibiotic synergy (26). Additionally, PAW has shown promising antibacterial properties (27, 28). However, its clinical utility is limited by pH-dependent activity (14).

Plasma is defined as the fourth state of matter, an ionized gas that, when used to treat aqueous samples, generates long-lived molecules such as ROS and reactive nitrogen species (RNS) (13), which have demonstrated efficacy against bacteria, fungi, and viruses in acidic environments (14). Research indicates that PAW, with a sufficiently low pH, converts superoxide anion radicals into hydroperoxyl radicals, which can penetrate cell membranes and disrupt intracellular components, exhibiting powerful membrane-permeabilizing effects (29). However, these also result in significant cytotoxicity due to non-specific oxidative damage to mammalian cells, thereby limiting their therapeutic window.

In contrast, NPAW (pH 7.4) maintains a stable composition of reactive oxygen and nitrogen species, including ·NO, ONOO⁻, and H₂O₂, which means that when the pH of the solution is above 4.8, superoxide anions do not convert extensively into hydroperoxyl radicals, thus avoiding pH-driven cytotoxicity (16, 30). Consequently, NPAW itself lacks direct bactericidal activity, consistent with prior observations. Previous studies have shown that short-term pre-treatment with PAW can enhance antibiotic sensitivity and promote antibiotic inactivation in methicillin-resistant *Staphylococcus aureus* (29). However, the combined effects of NPAW and antibiotics against bacteria have not been reported. In this study, we found that the combination of NPAW and polymyxin B enhances antibiotic efficacy.

Polymyxin primarily targets the lipopolysaccharides of the bacterial outer membrane, leading to cell-wall disruption and cell lysis. Bacteria can mitigate the effects of polymyxin through chromosomal mutations or gene recombination, altering lipopolysaccharide structures or enhancing biofilm formation, thereby reducing polymyxin accumulation and diminishing its antibacterial efficacy. Sun et al. (31) previously demonstrated that auranofin restores Polymyxin activity against CRAB by enhancing membrane permeability, suggesting that disrupting outer membrane integrity can improve polymyxin’s therapeutic effect. In our experiments, we observed that NPAW exacerbated the outer membrane damage induced by polymyxin B, evidenced by increased outer membrane permeability. This observation aligns with Rothwell et al., who attributed the membrane effects of PAW to RONS-mediated lipid peroxidation (12).

In our study, NPAW intensified the outer membrane damage caused by polymyxin B. This synergistic effect likely stems from RONS-mediated lipid peroxidation, which compromises cell membrane integrity and enhances polymyxin B’s access to lipid A targets. Furthermore, this interaction triggers an oxidative burst, leading to elevated intracellular ROS levels compared with polymyxin B monotherapy. Notably, excessive ROS production is associated with ATP depletion, indicating a potential vulnerability in polymyxin-resistant strains under standard treatment.

In summary, our findings advocate for utilizing NPAW as a promising adjunct to polymyxin B in combating MDR *A. baumannii* infections. This multifaceted approach has the potential to enhance therapeutic efficacy while mitigating resistance mechanisms, presenting a novel avenue for addressing these challenging infections.

### Conclusion

In conclusion, we report that the combination of NPAW with polymyxin B may be a viable alternative nebulizer treatment of pneumonia infected by polymyxin-resistant *A. baumannii* strains. This may confirm the potential efficacy of NPAW to treat clinical *A. baumannii*.

## Data Availability

The raw data have been deposited in the National Center for Biotechnology Information under the BioProject number PRJNA1304705.
